# Dataset of the COVID-19 post-lockdown survey conducted by GIPEyOP in Spain

**DOI:** 10.1016/j.dib.2021.107763

**Published:** 2021-12-24

**Authors:** Virgilio Pérez, Cristina Aybar, Jose M. Pavía

**Affiliations:** a GIPEyOP, Department of Applied Economics, University of Valencia, Spain; b GIPEyOP, UMMICS, Department of Applied Economics, University of Valencia, Spain

**Keywords:** SARS-CoV-2, New normal, Change of habits, Social distancing, Online surveys, Snowball method, Political assessment

## Abstract

2020 was a year marked by COVID-19, an infectious disease caused by the SARS-CoV-2 virus. Since the official beginning of the pandemic (March 2020), the authorities in Spain have been imposing significant restrictions (mainly on mobility) to stop the spread of the disease. In October 2020, the research group GIPEyOP (Elections and Public Opinion Research Group from the University of Valencia) conducted a survey to analyse whether the Spanish population has maintained or modified their habits and customs once the strict measures imposed in Spain during the onset of the pandemic were relaxed. This article describes the dataset collected, which is provided as an attachment. The dataset is made up of 196 variables, following elimination of those variables that could potentially identify the respondents to ensure their anonymity. Over 22 days, from September 23 to October 14, 2020, GIPEyOP collected 1755 valid responses. Respondents were contacted by chain or snowball sampling via email and social media and answered a self-administered web questionnaire consisting of 40 questions. amongst other uses, the resulting dataset can be (re)used to analyse whether the period of home confinement that Spaniards experienced between March and June 2020 has caused them to change their habits and customs, such as how often they do sport or go to bars or restaurants. The data also permit the study of whether there have been changes in the distribution of household chores by comparing three clearly differentiated moments (before confinement, during confinement and after confinement), what type of work (telework or face-to-face) the respondents would prefer or to know how the management of the crisis by govern authorities impacted on their votes preferences.

## Specifications Table

**Table:** 

Subject	Social Science, Sociology, Political Science, Health, Economy
Specific subject area	Social Science (general), Public opinion, Political Science, Health, Economics
Type of data	Table (spreadsheet)
How the data were acquired	Data were collected using a self-administered online questionnaire. The questionnaire is provided in Spanish (original) and English (translated) as a supplementary file (word format). A snowball or chain sampling method was used to recruit respondents.
Data format	Raw
Description of data collection	The survey was carried out at the beginning of autumn 2020, once the state of alarm decreed by the Spanish Government had ended, a measure implemented to stop the evolution of the COVID-19 pandemic. The survey data was collected over twenty-two days (between September 23 and October 14, 2020).
Data source location	Country: Spain
Data accessibility	Data file (spreadsheet) is supplied as supplementary material with this article.

## Value of the data

• This dataset offers information on various dimensions related to how the COVID-19 pandemic impacted on the Spanish population once a summer period with very relaxed measures had ended and a new COVID-19 wave was starting, with people having fresh in their memories a period of 99 days of home confinement just before summer.
• These data can be used by economists, sociologists, and political scholars to assess gender theories of behaviour within home in a scenario of the blurring of traditional gender roles.
• Economists could use these data to evaluate how the changes of habits have impacted on the economy.
• Political scientists could analyse how COVID-19 policies implemented by governments impacted on voting preferences.
• Psychologists may find these data useful to measure how the fear of being infected impacted on the preferences and habits of Spaniards when they spent time out of the house.
• This dataset is an example of how valuable information can be extracted from non-random samples.

## 1 Data Description

This document describes the data collected through a survey conducted between September 23 and October 14, 2020, a period in which SARS-CoV-2 coronavirus infections caused a second wave of contagions in Spain. The survey data file spreadsheet that accompanies this article consists of 1755 rows and 196 columns. Each row presents an individual's response to the questionnaire and each column represents each of the variables generated from a questionnaire made up of 40 questions. The questionnaire, available as supplementary material to this article in Spanish and translated into English, is divided into 9 sections: (i) demographic variables, including age, gender, and province of residence, amongst others; (ii) daily life, focusing on the characteristics of the residence in which the respondent resides; (iii) employment situation, made up of 8 questions about work or study environment; (iv) household chores; (v) fears and cares, about the health conditions (physical and mental) of the respondents and about their habits after lockdown; (vi) holidays (destinations where respondents have chosen to go on holiday, and methods used to manage the corresponding booking arrangements); (vii) social life, including questions about respondents' social habits; (viii) digital divide, providing information about the apps that respondents use on their smartphones, or the type and quality of the Internet connection, amongst other questions; (ix) political management of the pandemic and related to the electoral debate.

The dataset and the dictionary of variables are supplied as supplementary material. In this dataset (spreadsheet) two types of missing values can be distinguished: blank cells, corresponding to non-response, and cells with the value N/A (Not Applicable), which refer to those questions not applicable for those surveyed for whom a certain question did not need answering due to their answers to previous questions (see Table 1). Table 1 shows a brief description of the 196 variables available in the dataset. For reasons of space, the detail of the values for the PROV variable (Section 1), the Spanish province to which the respondent's municipality of residence belongs, is provided in Table A1 (Appendix file) and not in Table 1. Likewise, Table A2 (Appendix file) offers the response options for the variables R.VOTE (vote recall) and VOTE (vote intention), both in Section 9. This table contains the main political parties that stood in the 2019 general elections.

**Table 1:** Description of variables.

Section	Question code	Description	Values
		Respondent identification number	Number between 1 and 1755
		Time when the questionnaire was started / finished	Date and time

I	1001	Respondent's province of residence	See Table A1 (Appendix file)
	1002	Size of the municipality where the respondent resides	1. Less than 2000 inhabitants 2. Between 2001 and 10,000 3. Between 10,001 and 50,000 4. Between 50,001 and 100,000 5. Between 100,001 and 400,000 6. Between 400,001 and 1,000,000 7. More than 1,000,000 inhabitants
	1003	Gender of the respondent	1. Male 2. Female
	1004	Year of birthday	Number between 1919 and 2003
	1005	Highest education level achieved	1. Without studies 2. Primary education 3. Secondary education 4. Job training 5. Baccalaureate 6. University studies

II	2001	Do you live in the same home where you resided during the state of alarm (between 15 March and 21 June)?	1. Yes, it is my usual home. 2. Yes, although it is not my usual residence. 3. Yes, I would like to change, but I have no choice. 4. No, I have moved to another residence. 5. No, I have returned to my usual residence.
	2002	How many people do you live with?	1. None 2. One 3. Two 4. Three 5. Four 6. Five or more
	2002A	Could you please indicate the number of dependents you live with? *Note: only to be answered by respondents who did not choose 1 in question 2002.*	1. None 2. One 3. Two 4. Three 5. Four 6. Five or more
	2002B	Could you please indicate the number of high-risk people you live with? *Note: only to be answered by respondents who did not choose 1 in question 2002.*	1. None 2. One 3. Two 4. Three 5. Four 6. Five or more
III	3001	Comparison of the current employment situation with respect to that experienced before / during the state of alarm.	1. Same 2. Better 3. Worse
	3002	Respondent's employment situation.	1. I am a salaried employee and telework. 2. I am a salaried employee and I work outside the home. 3. I am a salaried employee and I combine telework and work outside the home. 4. I am self-employed and telework. 5. I am self-employed and I work outside the home. 6. I am self-employed and I combine telework and work outside the home. 7. I am self-employed with no possibility of practising my profession. 8. I am on an ERTE (Temporary Lay-off Plan). 9. I was fired after the state of alarm period. 10. I was fired during the state of alarm period. 11. Sick leave/pregnancy. 12. I am unemployed or on leave of absence. 13. Retired 14. Student 15. Unpaid work at home. 16. I work without a contract outside the home. 17. Other
	3003	Respondent's labour sector. *Note: only to be answered by respondents who chose 1, 2 or 3 in question 3002.*	1. Private sector 2. Public sector 3. Both sectors
	3003A1	Do you feel that your productivity at work has been affected since the end of the state of alarm? *Note: only to be answered by respondents who chose 1 to 6 in question 3002.*	1. Yes, I have a higher performance. 2. Yes, I have a lower performance. 3. No.
	3003A2	Do you think your work is threatened by this second wave of infections? *Note 1: only to be answered by respondents who chose 1 to 6 or 16 in question 3002.* *Note 2: you can indicate more than one option.*	1. Yes, because of a lack of economic activity due to the crisis. 2. Yes, due to staff cuts. 3. Yes, due to salary cuts. 4. Yes, because of having had to help in the family environment and having underperformed at work. 5. No, everything will stay the same.
	3002B	What is your experience of working at home after the end of the state of alarm? *Note 1: only to be answered by respondents who chose 1, 3, 4 or 6 in question 3002.* *Note 2: you can indicate more than one option.*	1. I am making better use of my time than in my place of work. 2. It is difficult to reconcile work and family life. 3. I wouldn't mind continuing to telework. 4. I prefer to commute to my place of work. 5. I would like to alternate between the two options. 6. I was already teleworking before the state of alarm.
	3002C1	Concern about the operation of the school year. *Note: only to be answered by respondents who chose 14 in question 3002.*	1. Yes, I am afraid of being in the classroom and catching the virus. 2. Yes, I am not sure how the classes will be taught. 3. Yes, I have not received enough information. 4. Yes, I do not have the conditions to study from home if necessary. 5. Yes, for other reasons. 6. I have no concerns.
	3002C2	How are you going to cover the costs of university studies? *Note: only to be answered by respondents who chose 14 in question 3002.*	1. My family. 2. I have started to work since the end of the state of alarm. 3. I was already working before the state of alarm started. 4. I have savings. 5. A scholarship. 6. I have applied for a scholarship due to lack of resources. 7. Others.

IV	4001	Weekly frequency of the corresponding task (14 tasks and 3 moments in time). *Note: tasks and moments in time are shown in*Table 2*.*	0 times to 7 times; No proceed to response
	4002	Outside help with housework before / after lockdown (weekly frequency).	0 times to 7 times; No proceed to response

V	5001	Fear of leaving the home	1. I have practically not gone out, but I am not afraid. 2. I have practically not gone out because I am afraid. 3. I go out alone to carry out basic tasks (walking the dog, shopping, work, care …), although I am afraid. 4. I go out alone to carry out basic tasks (walking the dog, shopping, work, care …), and I am not afraid. 5. I go out normally and I am not afraid. 6. I go out normally, but I am a bit afraid.
	5002	How well did you sleep compared to before the lockdown?	1. Same 2. Better 3. Worse
	5003	What hygiene measures do you usually take? *Note: you can indicate more than one option.*	1. I change my masque after its useful life. 2. I wear a masque, but I reuse it more than I should. 3. When I take off my masque, I am careful where I keep it. 4. I sanitise my hands whenever I touch something (public transport, coins…). 5. I take care of my immune system (food, vitamin supplements, physical exercise…). 6. I do not follow any special measures; I wear a masque out of obligation.

VI	6001	What did you do during your holidays last summer (2019)? *Note: you can indicate more than one option.*	1. Worked. I did not have any holiday time. 2. Worked and enjoyed a few days of holiday. 3. Did not travel due to lack of financial resources. 4. Went to the countryside, to my second residence. 5. Went to the beach, to my second residence. 6. Went to the mountains, to my second residence. 7. Went on a trip in Spain. 8. Went on a trip outside of Spain. 9. Others.
	6002	How much time have you had for holidays since the state of alarm ended?	1. I have had no holiday time. 2. Less than a week. 3. About fifteen days. 4. Between fifteen days and a month. 5. More than one month.
	6002A	What did you do during your holidays this summer (2020)? *Note 1: only to be answered by respondents who did not choose 1 in question 6002.* *Note 2: you can indicate more than one option.*	1. Did not travel due to lack of financial resources. 2. Did not travel due to economic uncertainty. 3. Did not travel due to fear of contagion. 4. Went to the countryside, to my second residence. 5. Went to the beach, to my second residence. 6. Went to the mountains, to my second residence. 7. Went on a trip in Spain. 8. Went on a trip outside of Spain. 9. Others.
	6002B	How have you managed to make arrangements for your holidays? *Note: only to be answered by respondents who chose 4 to 8 in question 6002A.*	1. I have not needed to make any arrangements. 2. By phone. 3. By Internet.
	6002C	Were you afraid of COVID-19 on your holiday? *Note 1: only to be answered by respondents who don't chose 4 to 8 in question 6002A.* *Note 2: you can indicate more than one option.*	1. Yes, people did not wear masks. 2. Yes, people did not keep a safe distance. 3. Yes, there was an outbreak in a nearby location. 4. Yes, in my local environment there was a contagion. 5. Yes, around me were high risk people. 6. I was afraid out of respect for the disease. 7. I was not afraid.

VII	7001	Have you changed your habits regarding the number of people you interact with? *Note: you can indicate more than one option.*	1. No, same as before. 2. Yes, I only socialise with the people I live with. 3. Yes, I only socialise with the people within my closest circle. 4. Yes, I have reduced the number of people I interact with.
	7002	Have you changed your habits outside your usual residence?	1. I only choose open spaces. 2. I might choose closed spaces as long as there is good ventilation. 3. I usually avoid closed spaces. 4. I do not restrict my choices.
	7003	How often did you used to go to a bar or restaurant before the state of alarm / since the state of alarm ended?	1. Every day 2. 5 or 6 days a week 3. 3 or 4 days a week 4. 1 or 2 days a week 5. Never
	7004	How often did you play sports before the state of alarm / since the state of alarm ended?	1. Every day 2. 5 or 6 days a week 3. 3 or 4 days a week 4. 1 or 2 days a week 5. Never

VIII	8001	Do you use a smartphone or the internet more? (for work / for personal issues) *Note: you can indicate more than one option.*	1. No, same as before 2. Yes, more video conferencing 3. Yes, more emails 4. Yes, more use of Twitter 5. Yes, more use of Facebook 6. Yes, more use of WhatsApp 7. Yes, more use of Telegram 8. Yes, more use of Instagram
	8002	Select the apps you used before the pandemic / currently use. *Note: you can indicate more than one option.*	1. Facebook 2. Instagram 3. WhatsApp 4. Telegram 5. Twitter 6. YouTube 7. E-mail (for personal use) 8. E-mail (to work) 9. Skype 10. Zoom 11. Teams 12. TikTok 13. Tinder 14. Others
	8003	What device do you usually use to connect to the internet?	1. Smartphone 2. Tablet 3. Laptop 4. PC 5. Smartwatch 6. Others
	8004	How do you prefer to communicate when you do not do so in person? (for work / for personal issues)	1. Telephone call using a landline 2. Telephone call using a mobile phone 3. Message written by WhatsApp or Telegram 4. Written email 5. Audio message by WhatsApp or Telegram 6. Videoconference (WhatsApp, Skype, Zoom …)
	8005	Do you usually have problems with your internet connection?	1. No. 2. Yes, the network goes down sometimes 3. Yes, the network crashes 4. Yes, the network is slow

IX	9001 9002	Valuation of the local/regional/national government in terms of management of the health/economic crisis (before summer/second wave).	0 (very bad) to 10 (very good)
	9003	Could you tell me which party you voted for in the last General Election?	1. List of parties in Table A2 (Appendix file) 2. Others 3. I was not old enough to vote 4. Abstention 5. I voted blank
	9004	If a congressional election were held today, which party would you vote for?	1. List of parties in Table A2 (Appendix file) 2. Others 3. I would not vote 4. I don't have the right to vote
		Time taken to complete the questionnaire	Numbers of seconds taken
		Time needed to complete Sections (I–IX)	Numbers of seconds taken

The mismatch between the number of questions (40) and the number of variables (196) comes from the fact that there are many questions for which more than a variable is extracted: (i) those questions in which the respondent could mark more than one answer have produced as many variables as there were answer options; (ii) questions in which the same topic was questioned at different moments in time; and (iii) the combination of both. This is the case of question 4001, about performing 14 household chores in 3 moments (before lockdown, during the lockdown, and after lockdown), giving rise to 42 variables (see Table 2). This question constitutes one of the central elements of the questionnaire and has helped, together with the data collected in [1], to carry out the research reported in [2] and [3].

**Table 2:** Weekly frequency (average number of days) of the respondent carrying out certain household chores before lockdown (BL), during lockdown (DL), and after lockdown (AL), by gender.

	Male			Female		
Household chores	BL	DL	AL	BL	DL	AL
Preparing midday meal	3.28	4.30	3.59	4.26	5.56	4.71
Cleaning the bathroom	1.37	1.92	1.36	2.57	3.59	2.79
Helping with children's homework	1.10	1.63	1.03	1.15	1.94	1.18
Playing with minors	1.84	2.05	1.77	1.97	2.36	2.01
Preparing the dinner	4.43	4.83	4.47	5.19	5.59	5.21
Bathing dependant persons	0.62	0.69	0.64	1.09	1.19	1.09
Leaving the house to look after other dependents	0.61	0.67	0.58	0.93	1.06	0.89
Washing up after meals	4.22	4.57	4.13	4.90	5.40	5.03
Dusting	1.34	1.79	1.27	2.05	2.88	2.15
Cleaning the floor	1.67	2.21	1.71	2.49	3.60	2.69
Going out for grocery shopping	2.65	2.26	2.54	2.56	1.48	2.18
Doing the washing	1.70	1.80	1.69	3.01	3.13	3.14
Ironing	0.66	0.63	0.61	1.17	1.14	1.18
Throwing out the rubbish	3.96	3.97	3.93	3.80	3.75	3.78

Table 3 provides information extracted from questions 7003 and 7004 (Section 7), referring to two specific customs and habits: (i) going to bars or restaurants and (ii) practising sports, before and after the state of alarm. The data provided in (i) was intensively exploited in [4] to study the impact of the pandemic on the Spanish restaurant sector. Table 4 offers information extracted from question 8001 (Section 8), referring to the increase in the use of new technologies, both in the workplace and in the family or personal environment. This information allows us to establish relationships between how much and in what context Spaniards use technologies, and how this influences at (tele)work. Table 5 presents a broad summary of the profile of the respondents in the survey. This explains the composition of the sample in terms of the main socio-economic-demographic characteristics, variables that in conjunction with R.VOTE (see Table A2 in Appendix file) are routinely used to correct biases of surveys in political studies.

**Table 3:** Comparison of habits and customs (before and after the state of alarm).

	Bars/Restaurants		Sport	
Category	Before	After	Before	After
Every day	116 (7.92)	39 (2.75)	295 (20.04)	230 (15.71)
5 or 6 days a week	119 (8.12)	28 (1.97)	231 (15.69)	188 (12.84)
3 or 4 days a week	340 (23.21)	111 (7.82)	427 (29.01)	348 (23.77)
1 or 2 days a week	785 (53.58)	729 (51.37)	343 (23.30)	472 (32.24)
Never	105 (7.17)	512 (36.08)	176 (11.96)	226 (15.44)

**Table 4:** Comparison of frequency of use of technological means (for work and for personal issues).

Category	For work	For personal issues
No, same as before	404 (23.02)	570 (32.48)
Yes, more video conferencing	563 (32.08)	508 (28.95)
Yes, more emails	431 (24.56)	184 (10.48)
Yes, more use of Twitter	43 (2.45)	195 (11.11)
Yes, more use of Facebook	40 (2.28)	238 (13.56)
Yes, more use of WhatsApp	324 (18.46)	707 (40.28)
Yes, more use of Telegram	40 (2.28)	118 (6.72)
Yes, more use of Instagram	34 (1.94)	231 (13.16)

**Table 5:** Respondent characteristics (*n* = 1755).

Characteristics	Category	Frequency (%)
Gender	Male	955 (54.42)
	Female	743 (42.34)
	*in blank*	57 (3.25)

Age (years)	< 20	6 (0.34)
	20–25	79 (4.50)
	26–30	117 (6.67)
	31–35	105 (5.98)
	36–40	144 (8.21)
	41–45	175 (9.97)
	46–50	184 (10.48)
	51–55	190 (10.83)
	56–60	220 (12.54)
	61–65	201 (11.45)
	66–70	149 (8.49)
	>70	151 (8.60)
	*in blank*	34 (1.94)

Employment situation	I am a salaried employee and telework. I am a salaried employee and I work outside the home. I am a salaried employee and I combine telework and work outside the home. I am self-employed and telework. I am self-employed and I work outside the home. I am self-employed and I combine telework with work outside the home. I am self-employed with no possibility of practising my profession. I am on an ERTE. I was fired after the state of alarm period. I was fired during the state of alarm period. Sick leave/pregnancy I am unemployed or on leave of absence. Retired Student Unpaid work at home I work without a contract outside my home. Other *in blank*	137 (7.81) 538 (30.66) 204 (11.62) 22 (1.25) 68 (3.87) 42 (2.39) 9 (0.51) 19 (1.08) 18 (1.03) 14 (0.80) 19 (1.08) 97 (5.53) 371 (21.14) 52 (2.96) 21 (1.20) 10 (0.57) 49 (2.79) 65 (3.70)

Education	Without studies	1 (0.06)
	Primary education	46 (2.62)
	Secondary education	63 (3.59)
	Job training	147 (8.38)
	Baccalaureate	187 (10.66)
	University studies *in blank*	1273 (72.54) 38 (2.17)

Residence municipality size (inhabitants)	Less than 2000 inhabitants	57 (3.25)
	Between 2001 and 10,000	192 (10.94)
	Between 10,001 and 50,000	379 (21.60)
	Between 50,001 and 100,000	189 (10.77)
	Between 100,001 and 400,000	225 (12.82)
	Between 400,001 and 1,000,000	352 (20.06)
	More than 1,000,000 de inhabitants *in blank*	290 (16.52) 71 (4.05)

## 2 Experimental Design, Materials and Methods

### 2.1 Data collection

In early 2020, the world was exposed to an extreme threat, that of the SARS-CoV-2 coronavirus health crisis. On 14 March, the government of Spain imposed, through a Royal Decree, a series of mandatory restrictive measures [5]. One of these measures was strict home confinement, which lasted 99 days. The favourable evolution of the pandemic allowed the authorities to lift the restrictions previously imposed from the end of spring, with life returning to what is generally referred to as the new normal [6], a situation in which the strict measures imposed at the beginning of the pandemic have been relaxed, but some limitations remain in place. However, the impact of the health crisis on Spanish society has led to a substantial change in habits and customs, for example, in the way people relate to each other or how public spaces are used and their frequency of use, in particular the use of closed spaces, such as bars and restaurants [4]. The data described in the present study show the responses of the Spanish population to a series of contextualized questions in a new period marked by the end of home confinement (which lasts more than three months) and after a summer in which the measures to contain the spread of the virus had been relaxed; just in a moment when Spain was seeing to have the highest number of people infected by COVID-19 of any European country [7].

Between September 23 and October 14, 2020, the Research Group on Electoral Processes and Public Opinion of the University of Valencia (GIPEyOP) prepared a survey to collect information from different social strata related to this context. The information gathered enables the comparison of the valuation and perception of Spaniards at different times of the pandemic and on topics as varied as sports practice, the use of mobile devices and the internet, evaluation of government officials and their management of the health and economic crisis, or the involvement of different members of the family unit in housework.

The survey, organised into nine blocks or sections, collected 1755 valid responses through a snowball sample design, initiated from a file of GIPEyOP collaborators. GIPEyOP collaborators are people who selflessly participate with the research group by voluntarily answering and forwarding, at their convenience, the surveys generated by GIPEyOP. When we finish an investigation, a report is sent to them with the results obtained, in gratitude for their collaboration. If a person wishes to be part of this group of collaborators, they must fill in the form available on the group website <gipeyop.uv.es>. The link to this form is also available at the end of all our surveys to enrol more collaborators. Of course, a collaborator can unsubscribe at any time, via personal communication or by filling in another form available on the GIPEyOP website.

The survey distribution process starts by sending by email a message to the GIPEyOP collaborators’ list. Included in this message is a URL through which to access the online survey. They are asked to fill in the survey and to share it with their contacts. The forwarding of the survey is very simple to carry out, since the collaborators, in addition to completing the survey, can forward the received message to their contacts. But not only that. They can also share the URL with their acquaintances using social networks, with WhatsApp, Facebook, and Twitter being the most used. The survey has specific utilities to do that. In this way, starting from the initial list of collaborators, we managed to get the survey to a much larger segment of the population.

The URL that gave access to the survey was accompanied by the following message: “From the GIPEyOP research group of the University of Valencia we are studying the effects of the COVID19 crisis. We ask you for 10 min of your time to answer the survey and also that, please, share it with people over 17 in your environment. We appreciate that you disseminate the survey through social networks and amongst your contacts. The success of the research depends on you, and the variety and amount of information that we can collect. Thank you”. In this way, the receiver of the link decided whether to access the questionnaire and/or resend it at that time, leave it for later or discard it definitively.

As mentioned above, the snowball technique was used to select the sample. This non-probabilistic method does not guarantee the representativeness of the sample, amongst other issues, the sample obtained is partially conditioned to the place of work or residence of the person/people who initiate the process. However, this procedure has some advantages over other sampling techniques: (i) it is an inexpensive and simple process, which has been described in some detail in the previous paragraph; (ii) it makes it possible to exploit the possibilities offered by new information technologies, mainly virtual social networks; (iii) it requires few human resources since interviewers are not necessary and the interviewed subjects themselves help to enrol new respondents; (iv) makes it possible to sample populations that are difficult to access [8], [9]. But not only that. This technique allows, unlike other questionnaires that are repeated over time, to compare opinions, habits, and feelings of roughly similar groups at different moments in time. Furthermore, despite the biases in the data collected, when conditional inferences are made, the results of the modelling usually lead to conclusions equivalent to those obtained with representative samples [10].

To analyse the survey data, the individual responses obtained are weighted using post-stratification/calibration techniques to correct for biases in the collected sample [11]. To do that, we use two-class calibration approaches when we consider two variables to compute the sampling weights and marginal calibration (post-stratification) approaches when either one or more than two variables are employed. In our reports and models, we typically combine, in some cases, two or more of the following variables: province of residence, habitat size, gender, age, and education level. In other cases, we use the combination of the variables R.VOTE (party voted in last elections) and the province of residence. With these methods, we can compensate for the over-representation of some provinces or sociodemographic profiles in the sample.

Each of the questionnaires received was subjected to an intense filtering process to select only those questionnaires with minimum requirements in quality (internal consistency) and quantity of the available information. On the one hand, those questionnaires that did not contain a minimum number of responses were discarded. For example, as a rule, all samples that did not meet Section 3 were discarded. On the other hand, consistency tests were used, crossing pairs of variables, such as the size of the habitat and the province of residence. These actions resulted in the elimination of 233 responses (11.7% of the total). The validated dataset contains, as indicated above, a total of 1755 observations in 196 variables.

### 2.2 Value of the data

As previously mentioned, the survey is structured in nine sections or thematic blocks. The information collected in the first part of the study helps to define the social and demographic profile of the respondents in the survey, information that is of relevance when analysing the results. The questions in the second section (daily life) help assess, amongst other issues, whether Spaniards have modified their habits in terms of caring for dependant people or people at high risk of contagion, particularly important considering the high mortality that SARS-CoV-2 caused in the elderly [12]. Section 3 of the survey is dedicated to the respondent's work/educational environment and helps identify how, and to what extent, the period of confinement has modified how Spaniards work/study, and whether it has prompted a transition period towards a new paradigm marked by teleworking and online education [13], [14]. In both Sections 2 and 3 some questions enable the perception of the respondent at the time the survey was conducted (October 2020) to be compared with their perception during the period of confinement (second quarter of 2020, when a further survey was conducted [1]). The feelings that a person who works may have might be different from that of a student, a retiree, or someone unemployed with limited possibilities of finding work. In this sense, it is important to know how confinement affected studies or work performance, as well as to identify what new habits and customs are likely to remain once this exceptional situation has ended.

Section 4 focuses on analysing the completion of certain household chores (before, during, and after confinement). This block of questions reveals whether the situation of forced cohabitation changed the usual way of distributing chores related to the home and the care of minors or dependant elderly and whether, once the confinement period ended, these possible changes have been maintained or, on the contrary, things have returned to the way they were, or perhaps they depend on some co-variables, such as the habitat size [3] or the modality of work (telework or face-to-face) [15].

Section 5 of the survey focuses on three aspects related to the respondent's health: (i) fear of leaving the house; (ii) sleep disorders; and (iii) hygienic-sanitary measures. Studies have been published throughout the pandemic that demonstrates the relationship between the issues raised in this block and protection against the coronavirus [16]. Knowing whether these possible disorders have disappeared since the end of the state of alarm or whether they have been reduced may be important when considering the need for the implementation of therapeutic measures and for establishing certain action protocols.

Section 6 is orientated towards analysing decisions that the respondents made regarding their summer holidays of 2019 (before the pandemic) and 2020 (after the lockdown). The questions posed provide information on how the customs and habits of Spanish society have changed in this regard, making these data especially interesting for implementing new business models and for all agents related to the tourism sector, a sector severely affected by the pandemic [17].

Section 7 of the questionnaire includes questions related to certain social and consumption customs and habits, such as the frequency with which the respondents practised/practise sports or the regularity with which they frequented/frequent bars and restaurants (before and after the state of alarm). These types of questions help identify whether the behaviour patterns of Spaniards have been affected by the extreme situation experienced in 2020 and whether these possible changes will lead to transformations that may require certain sectors to adapt, such as the hospitality sector is doing, to guarantee their survival. In Madrid, for example, the limitations on mobility for so many months and the fear of contagion in closed spaces have meant that, with the support of the local government, bars and restaurants have had to reinvent themselves, adding/expanding outdoor terraces to adapt to the new demands of their customers [4].

Section 8 is dedicated to the use of the internet and applications on mobile devices, both in the workplace and in personal time. These questions help to identify whether the way Spaniards used to communicate has changed because of confinement and whether the technological resources currently available are adequate and sufficient. The emergence of teleworking has led to a significant increase in cyberattacks, both in the public and private sectors [18], posing major business challenges while generating new jobs and business opportunities.

In the last section of the survey, four political profile questions are asked that give an insight into how the population values how government officials have managed the health and economic crisis [19] and how this impact on their vote preferences. Specifically, it assesses how effectively the respondents think the government has managed the situation at local, regional, and national levels, comparing two moments in time: before the state of alarm was decreed and in the moment of conducting the survey, just when the second wave of infections began in Spain. Respondents are also asked which political party they voted for in the last election and what their choice would be if elections were held at present. By crossing these responses with the other variables, relationships can be established between political ideology, sociodemographic variables, and perceptions related to work and household conditions.

## Ethics Statements

At the beginning of the questionnaire, participants were informed that the survey was anonymous, voluntary and confidential, as established in the current regulations on Personal Data Protection and guarantee of digital rights. It was also indicated that the conclusions drawn from the survey would only be presented in aggregate form.

## CRediT authorship contribution statement

**Virgilio Pérez:** Data curation, Investigation, Visualization, Writing – original draft, Writing – review & editing. **Cristina Aybar:** Data curation, Funding acquisition, Investigation, Software, Validation, Visualization, Writing – original draft, Writing – review & editing. **Jose M. Pavía:** Conceptualization, Funding acquisition, Investigation, Project administration, Resources, Supervision, Validation, Writing – review & editing.

## Declaration of Competing Interest

The authors declare that they have no known competing financial interests or personal relationships that could have influenced the work reported in this paper.

## Appendix Supplementary materials

Image, application 1

Image, application 2

Image, application 3

Image, application 4

Image, application 5
