# Supplementary material for: Dataset of the COVID-19 post-lockdown survey conducted by GIPEyOP in Spain
Source: Data Brief. 2021 Dec 24;40:107763. doi: 10.1016/j.dib.2021.107763 (PMC8704783; doi:10.1016/j.dib.2021.107763)
Supplement: Supplementary file 1 [file mmc1.docx]

**Appendix**

**Table A1**

Provinces of Spain.

| **Province** | **Province** |
| --- | --- |
| 1. A Coruña 2. Albacete 3. Alicante/Alacant 4. Almería 5. Araba/Álava 6. Asturias 7. Ávila 8. Badajoz 9. Barcelona 10. Bizkaia 11. Burgos 12. Caceres 13. Cádiz 14. Cantabria 15. Castellón/Castelló 16. Ceuta 17. Ciudad Real 18. Córdoba 19. Cuenca 20. Gipuzkoa 21. Girona 22. Granada 23. Guadalajara 24. Huelva 25. Huesca 26. Illes Balears | 1. Jaén 2. La Rioja 3. Las Palmas 4. León 5. Lleida 6. Lugo 7. Madrid 8. Málaga 9. Melilla 10. Murcia 11. Navarra 12. Ourense 13. Palencia 14. Pontevedra 15. Salamanca 16. Santa Cruz de Tenerife 17. Segovia 18. Sevilla 19. Soria 20. Tarragona 21. Teruel 22. Toledo 23. Valencia/València 24. Valladolid 25. Zamora 26. Zaragoza |

**Table A2**

Political parties in 2019 General Elections

| **Political party** | **Abbreviation** |
| --- | --- |
| Partido Socialista Obrero Español  Partido Popular  VOX  Unidas Podemos  Ciudadanos  Esquerra Republicana de Catalunya  En comú podem  Junts per Catalunya - JUNTS  Euzko Alderdi Jeltzalea - Partido Nacionalista Vasco  Más País - EQUO  Euskal Herria Bildu  Candidatura de Unidad Popular - PR  Més - Compromís  Coalición Canaria – Nueva Canarias  Bloque Nacionalista Galego  Navarra Suma | PSOE  PP  VOX  UP  Cs  ERC  ECP  JxCAT - JUNTS  EAJ - PNV  MP – EQUO  EH Bildu  CUP – PR  Més - Compromís  CC- NC  BNG  N+ |
