# Supplementary material for: Dataset of the COVID-19 post-lockdown survey conducted by GIPEyOP in Spain
Source: Data Brief. 2021 Dec 24;40:107763. doi: 10.1016/j.dib.2021.107763 (PMC8704783; doi:10.1016/j.dib.2021.107763)
Supplement: Supplementary file 2 [file mmc2.pdf]

Encuesta COVID-19, otoño

Participar en esta encuesta es **voluntario** y **confidencial** (LO 3/2018 de Protección de Datos Personales y Garantía de los Derechos Digitales). Todas las respuestas son anónimas y las conclusiones serán presentadas de forma agregada. La encuesta permanecerá abierta hasta el **15 de octubre**.

Podrás evitar responder a cualquier pregunta que desees o para la que no conozcas la respuesta. El tiempo estimado para contestar la encuesta es de **10 minutos**.

Para avanzar en la encuesta ve al final de la página y pulsa Siguiente.

Gracias a la colaboración ciudadana, desde GIPEyOP estamos consiguiendo mejorar las metodologías de análisis y con ello poder ser de utilidad, ofreciendo resultados de **calidad**, **objetivos** y **creíbles**. Queremos que nos ayudes a seguir avanzando en esa dirección. Para obtener más información, conocer las condiciones en que se enmarca esta encuesta o sobre sus responsables, pincha **aquí**.

Agradeceríamos INVITARAS A TUS CONTACTOS A PARTICIPAR EN LA ENCUESTA, reenviándoles el enlace o compartiéndolo en tus redes sociales.

Hay 40 preguntas en la encuesta

Sección I (variables demográficas)

(S1001) Por favor, selecciona la provincia en la que resides en estos momentos

Por favor seleccione **sólo una** de las siguientes opciones:

- A Coruña
- Albacete
- Alicante/Alacant
- Almería
- Araba/Álava
- Asturias
- Ávila
- Badajoz
- Barcelona
- Bizkaia
- Burgos
- Cáceres
- Cádiz
- Cantabria
- Castellón/Castelló
- Ceuta
- Ciudad Real
- Córdoba
- Cuenca
- Gipuzkoa
- Girona
- Granada
- Guadalajara
- Huelva
- Huesca
- Illes Balears
- Jaén
- La Rioja
- Las Palmas
- León
- Lleida
- Lugo
- Madrid
- Málaga
- Melilla
- Murcia
- Navarra
- Ourense
- Palencia
- Pontevedra
- Salamanca
- Santa Cruz de Tenerife
- Segovia
- Sevilla
- Soria
- Tarragona
- Teruel
- Toledo
- Valencia/València
- Valladolid
- Zamora
- Zaragoza

(S1002) ¿Podrías decirnos el tamaño del municipio en el que resides?

Por favor seleccione **sólo una** de las siguientes opciones:

- Menos de 2.000 habitantes
- Entre 2.001 y 10.000
- Entre 10.001 y 50.000
- Entre 50.001 y 100.000
- Entre 100.001 y 400.000
- Entre 400.001 y 1.000.000
- Más de 1.000.000 de habitantes

(S1003) Sexo

Por favor seleccione **sólo una** de las siguientes opciones:

- Hombre
- Mujer

(S1004) ¿Podrías indicar tu año de nacimiento?

(introduce cuatro cifras)

Su respuesta debe estar entre 1919 y 2003

Sólo un valor entero puede ser introducido en este campo.

Por favor, escriba su respuesta aquí:

- ________

(S1005) ¿Podrías decirnos tus estudios?

Por favor seleccione **sólo una** de las siguientes opciones:

- Sin estudios
- Primarios
- Secundarios
- Formación profesional
- Bachillerato
- Universitarios

Sección II (vida cotidiana)

(S2001) En estos momentos, el lugar donde vives ¿es la misma residencia en la que estuviste confinado/a durante el estado de alarma (15 de marzo a 21 de junio)?

Por favor seleccione **sólo una** de las siguientes opciones:

- Sí, es mi vivienda de siempre
- Sí, aunque no es mi residencia habitual
- Sí, necesitaría cambiar, pero no tengo alternativa
- No, me he cambiado a otra residencia
- No, he vuelto a mi residencia habitual

(S2002) ¿Con cuántas personas convives, sin incluirte?

Por favor seleccione **sólo una** de las siguientes opciones:

- Ninguna
- Una
- Dos
- Tres
- Cuatro
- Cinco o más

(S2002A) ¿Podrías indicar el número de personas dependientes con las que convives?

(enferm@s, menores, personas disfuncionales, …)

Sólo conteste esta pregunta si se cumplen las siguientes condiciones:

La respuesta NO fue 'Ninguna' en la pregunta (S2002)

Por favor seleccione **sólo una** de las siguientes opciones:

- Ninguna
- Una
- Dos
- Tres
- Cuatro
- Cinco o más

(S2002B) ¿Podrías indicar el número de personas de mayor riesgo con las que convives?

(mayores, embarazadas, sanitari@s, policías, cajer@s, …)

Sólo conteste esta pregunta si se cumplen las siguientes condiciones:

La respuesta NO fue 'Ninguna' en la pregunta (S2002).

Por favor seleccione **sólo una** de las siguientes opciones:

- Ninguna
- Una
- Dos
- Tres
- Cuatro
- Cinco o más

Sección III (situación laboral)

(S3001) ¿Podrías marcar cómo es tu situación laboral? Comparado con…

Por favor, seleccione la respuesta apropiada para cada concepto:

	el estado de alarma	antes del estado de alarma
**Igual**		
**Mejor**		
**Peor**		

(S3002) ¿Podrías marcar en cuál de los siguientes perfiles de actividad te encuentras en estos momentos?

Por favor seleccione **sólo una** de las siguientes opciones:

- Soy asalariad@ y teletrabajo
- Soy asalariad@ y salgo de casa para trabajar
- Soy asalariad@ y combino teletrabajo y trabajo fuera de casa
- Soy autónom@ y teletrabajo
- Soy autónom@ y salgo de casa para trabajar
- Soy autónom@ y combino teletrabajo y trabajo fuera de casa
- Soy autónom@ sin posibilidad de ejercer mi profesión
- Estoy en un ERTE
- Me despidieron después del periodo de estado de alarma
- Me despidieron durante el periodo de estado de alarma
- Estoy de baja por enfermedad/embarazo
- Parad@ o en excedencia
- Jubilad@
- Soy estudiante
- Realizo trabajo no remunerado en el hogar
- Trabajo sin contrato fuera de casa
- Otro

(S3003) Completa la frase: trabajo en…

Sólo conteste esta pregunta si se cumplen las siguientes condiciones:

La respuesta fue 'Soy asalariad@ y teletrabajo' o 'Soy asalariad@ y salgo de casa para trabajar' o 'Soy asalariad@ y combino teletrabajo y trabajo fuera de casa' en la pregunta (S3002).

Por favor seleccione **sólo una** de las siguientes opciones:

- …el sector privado
- …el sector público
- …ambos sectores

(S3003A1) Respecto al estado de alarma, ¿sientes que tu rendimiento en el trabajo se ha visto afectado tras el final del estado de alarma?

Sólo conteste esta pregunta si se cumplen las siguientes condiciones:

La respuesta fue 'Soy asalariad@ y teletrabajo' o 'Soy asalariad@ y salgo de casa para trabajar' o 'Soy asalariad@ y combino teletrabajo y trabajo fuera de casa' o 'Soy autónom@ y teletrabajo' o 'Soy autónom@ y salgo de casa para trabajar' o 'Soy autónom@ y combino teletrabajo y trabajo fuera de casa' o 'Trabajo sin contrato fuera de casa' en la pregunta (S3002).

Por favor seleccione **sólo una** de las siguientes opciones:

- Sí, mayor rendimiento
- Sí, peor rendimiento
- No

(S3003A2) ¿Crees que tu trabajo está amenazado por esta segunda ola de contagios?

(puedes señalar más de una opción)

Sólo conteste esta pregunta si se cumplen las siguientes condiciones:

La respuesta fue 'Soy asalariad@ y teletrabajo' o 'Soy asalariad@ y salgo de casa para trabajar' o 'Soy asalariad@ y combino teletrabajo y trabajo fuera de casa' o 'Soy autónom@ y teletrabajo' o 'Soy autónom@ y salgo de casa para trabajar' o 'Soy autónom@ y combino teletrabajo y trabajo fuera de casa' o 'Trabajo sin contrato fuera de casa' en la pregunta (S3002).

Por favor, marque las opciones que correspondan:

- Sí, por falta de actividad económica debido a la crisis
- Sí, por recortes de plantilla
- Sí, por recortes de salario
- Sí, por haber tenido que ayudar en el entorno familiar y haber rendido menos en el trabajo
- No, todo seguirá más o menos igual

(S3002B) ¿Cuál está siendo tu experiencia trabajando en casa tras el fin del estado de alarma?

(puedes señalar más de una opción)

Sólo conteste esta pregunta si se cumplen las siguientes condiciones:

La respuesta fue 'Soy asalariad@ y combino teletrabajo y trabajo fuera de casa' o 'Soy autónom@ y combino teletrabajo y trabajo fuera de casa' o 'Soy asalariad@ y teletrabajo' o 'Soy autónom@ y teletrabajo' en la pregunta (S3002).

Por favor, marque las opciones que correspondan:

- Estoy aprovechando más el tiempo que en mi centro de trabajo
- Es difícil conciliar/compaginar la vida laboral y la vida familiar
- No me importaría seguir teletrabajando
- Prefiero desplazarme al centro de trabajo
- Me gustaría alternar ambas opciones
- Ya teletrabajaba antes del estado de alarma

(S3002C1) ¿Sientes inquietud por cómo transcurrirá el curso 20-21?

Sólo conteste esta pregunta si se cumplen las siguientes condiciones:

La respuesta fue 'Soy estudiante' en la pregunta (S3002).

Por favor seleccione **sólo una** de las siguientes opciones:

- Sí, siento temor de estar en las aulas y contagiarme
- Sí, no sé bien cómo se van a dar las clases
- Sí, no he recibido suficiente información
- Sí, no dispongo de condiciones para realizarlo desde casa si fuera necesario
- Sí, por otros motivos
- No tengo ninguna inquietud

(S3002C2) ¿Cómo vas a sufragar los gastos de los estudios universitarios?

(puedes señalar más de una opción)

Sólo conteste esta pregunta si se cumplen las siguientes condiciones:

La respuesta fue 'Soy estudiante' en la pregunta (S3002).

Por favor, marque las opciones que correspondan:

- Mi familia
- Estudio y he comenzado a trabajar después del estado de alarma
- Trabajaba y estudiaba antes del estado de alarma
- Ahorros que tengo
- Una beca
- He solicitado una beca para este curso por falta de recursos
- Otros

Sección IV (tareas del hogar)

(S4001) En cada actividad, deja la casilla en blanco si no procede o señala un 0, 1, 2, 3, 4, 5, 6 o 7 según el número de días por semana que realizabas/realizas las siguientes actividades

	Antes del confinamiento	Durante el confinamiento	Actualmente
Preparar comida a mediodía	☐	☐	☐
Limpiar el baño	☐	☐	☐
Ayudar en tareas escolares	☐	☐	☐
Jugar con menores	☐	☐	☐
Preparar cenas	☐	☐	☐
Bañar a personas dependientes	☐	☐	☐
Salir de casa para atender a otras personas	☐	☐	☐
Fregar después de las comidas	☐	☐	☐
Limpiar el polvo	☐	☐	☐
Limpiar el suelo	☐	☐	☐
Comprar en tiendas de comida	☐	☐	☐
Lavar la ropa	☐	☐	☐
Planchar	☐	☐	☐
Tirar la basura	☐	☐	☐

(S4002) ¿Cuántos días de la semana disponías/dispones de ayuda externa para realizar las tareas del hogar?

	Frecuencia semanal
Antes del confinamiento	☐
Después del confinamiento	☐

Sección V (miedos y cuidados)

(S5001) Hablemos sobre salir de la vivienda después del confinamiento

Por favor seleccione **sólo una** de las siguientes opciones:

- Prácticamente no he salido, pero no tengo ningún miedo
- Prácticamente no he salido, por miedo
- Salgo solo a realizar tareas básicas (pasear al perro, compras, trabajo, cuidados…) aunque con temor
- Salgo solo a realizar tareas básicas (pasear al perro, compras, trabajo, cuidados…) sin miedo
- Salgo con normalidad y no tengo ningún miedo
- Salgo con normalidad pero tengo algún miedo

(S5002) En general, respecto a antes del inicio de esta crisis que estamos viviendo, ¿cómo estás durmiendo?

Por favor seleccione **sólo una** de las siguientes opciones:

- Igual
- Mejor
- Peor

(S5003) En la situación actual, ¿cuál o cuáles de las siguientes medidas estás siguiendo?

(puedes señalar más de una opción)

Por favor, marque las opciones que correspondan:

- Cambio mascarilla tras su periodo de vida útil
- Uso mascarilla pero la reutilizo más de lo que debiera
- Cuando me la quito tengo cuidado de dónde la guardo
- Me higienizo las manos siempre que toco algo (en el autobús, metro, monedas, billetes…)
- Me cuido el sistema inmunológico (alimentación, suplementos, ejercicios…)
- No sigo ninguna medida especial, llevo mascarilla por obligación

Sección VI (vacaciones)

(S6001) ¿Qué hiciste el verano del año pasado, 2019?

(puedes señalar más de una opción)

Por favor, marque las opciones que correspondan:

- Trabajar, no tuve ningún día de vacaciones
- Trabajar y disfrutar de unos días de vacaciones
- No desplazarme por falta de recursos económicos
- Ir al campo, a mi segunda residencia
- Ir a la playa, a mi segunda residencia
- Ir a la montaña, a mi segunda residencia
- Salir de viaje por España
- Salir de viaje fuera de España
- Otros

(S6002) ¿Cuánto tiempo has tenido de vacaciones después del estado de alarma?

Por favor seleccione **sólo una** de las siguientes opciones:

- No he tenido vacaciones
- Menos de 1 semana
- Alrededor de 15 días
- Entre 15 días y 1 mes
- Más de 1 mes

(S6002A) ¿Y qué has hecho durante los días de vacaciones que has tenido después del estado de alarma?

(puedes señalar más de una opción)

Sólo conteste esta pregunta si se cumplen las siguientes condiciones:

La respuesta fue 'Más de 1 mes' o 'Entre 15 días y 1 mes' o 'Alrededor de 15 días' o 'Menos de 1 semana' en la pregunta (S6002).

Por favor, marque las opciones que correspondan:

- No desplazarme por falta de recursos económicos
- No desplazarme por la incertidumbre económica
- No desplazarme por miedo al contagio
- Ir al campo, a mi segunda residencia
- Ir a la playa, a mi segunda residencia
- Ir a la montaña, a mi segunda residencia
- Salir de viaje por España
- Salir de viaje fuera de España
- Otros

(S6002B) ¿Cómo has gestionado las vacaciones?

Sólo conteste esta pregunta si se cumplen las siguientes condiciones:

La respuesta fue 'Ir al campo, a mi segunda residencia' o 'Ir a la playa, a mi segunda residencia' o 'Ir a la montaña, a mi segunda residencia' o 'Salir de viaje por España' o 'Salir de viaje fuera de España' en la pregunta (S6002A).

Por favor seleccione **sólo una** de las siguientes opciones:

- No he necesitado realizar ninguna gestión
- Por teléfono
- Por internet

(S6002C) ¿Has tenido miedo ante la Covid en tus vacaciones?

(puedes señalar más de una opción)

Sólo conteste esta pregunta si se cumplen las siguientes condiciones:

La respuesta fue 'Ir al campo, a mi segunda residencia' o 'Ir a la playa, a mi segunda residencia' o 'Ir a la montaña, a mi segunda residencia' o 'Salir de viaje por España' o 'Salir de viaje fuera de España' en la pregunta (S6002A).

Por favor, marque las opciones que correspondan:

- Sí, la gente no llevaba la mascarilla
- Sí, la gente no mantenía la distancia de seguridad
- Sí, hubo un brote en la localidad
- Sí, en mi entorno hubo un contagio
- Sí, a mi alrededor tengo personas de alto riesgo
- He tenido miedo por el respeto que me produce la enfermedad
- No he tenido miedo

Sección VII (vida social)

(S7001) Respecto al número de personas con las que te relacionas, ¿has modificado tus hábitos como consecuencia de la COVID?

(puedes señalar más de una opción)

Por favor, marque las opciones que correspondan:

- No, igual que antes
- Sí, sólo me relaciono con las personas con las que convivo
- Sí, sólo me relaciono con mi entorno más próximo
- Sí, he reducido el número de personas con las que me relaciono

(S7002) ¿Y has modificado los hábitos cuando sales de tu residencia habitual?

Por favor seleccione **sólo una** de las siguientes opciones:

- Sólo elijo espacios abiertos
- Puedo elegir espacios cerrados siempre que haya buena ventilación
- Suelo evitar los espacios cerrados
- No tengo restricciones

(S7003) ¿Con qué frecuencia solías/sueles ir a un bar o a un restaurante?

Por favor, selecciones la respuesta apropiada para cada concepto:

	Antes del estado de alarma	Después del estado de alarma
**Todos los días de la semana**		
**5 o 6 días a la semana**		
**3 o 4 días a la semana**		
**1 o 2 días a la semana**		
**Ningún día**		

(S7004) ¿Con qué frecuencia salías/sales ir a pasear o a hacer deporte?

Por favor, selecciones la respuesta apropiada para cada concepto:

	Antes del estado de alarma	Después del estado de alarma
**Todos los días de la semana**		
**5 o 6 días a la semana**		
**3 o 4 días a la semana**		
**1 o 2 días a la semana**		
**Ningún día**		

Sección VIII (brecha digital)

(S8001) Comparado con antes del inicio de la pandemia, ¿has aumentado el uso de internet y/o móvil?

(puedes señalar más de una opción)

	Por trabajo	Por cuestiones personales
No, igual que antes		
Sí, más videoconferencias		
Sí, más correos electrónicos		
Sí, más uso de Twitter		
Sí, más uso de Facebook		
Sí, más uso de WhatsApp		
Sí, más uso de Telegram		
Sí, más uso de Instagram		

(S8002) De la siguiente lista, ¿podrías marcar las aplicaciones que usas/usabas?

(puedes señalar más de una opción)

	antes de la pandemia	actualmente
Facebook		
Instagram		
WhatsApp		
Telegram		
Twitter		
YouTube		
Correo electrónico personal		
Correo electrónico trabajo		
Skype		
Zoom		
Tams		
TikTok		
Tinder		
Otras		

(S8003) ¿Qué dispositivo utilizas más habitualmente para internet?

Por favor seleccione **sólo una** de las siguientes opciones:

- Teléfono móvil
- Tableta
- Ordenador portátil
- PC fijo
- Reloj electrónico
- Otros dispositivos

(S8004) En general, ¿cómo prefieres comunicarte cuando no lo haces en persona?

Por favor, selecciones la respuesta apropiada para cada concepto:

	Por trabajo	Por motivos personales
**Llamada telefónica con teléfono fijo**		
**Llamada telefónica con móvil**		
**Mensaje escrito WhatsApp o Telegram**		
**Mensaje escrito por correo electrónico**		
**Mensaje de audio por WhatsApp o Telegram**		
**Videoconferencias WhatsApp, Skype, Zoom, etc.**		

(S8005) ¿Sueles tener problemas con internet?

Por favor seleccione **sólo una** de las siguientes opciones:

- No
- Sí, se cae la red en ocasiones
- Sí, se colapsa la red
- Sí, la venta en ocasiones

Sección IX (gestión política)

(S9001) En una escala de 0 a 10, ¿qué nota pondrías a la gestión de la pandemia antes del verano por parte de...?

Por favor, seleccione la respuesta apropiada para cada concepto:

	gestión sanitaria												gestión económica											
	No sabe / No contesta	0	1	2	3	4	5	6	7	8	9	10	No sabe / No contesta	0	1	2	3	4	5	6	7	8	9	10
**Gobierno de España**																								
**Gobierno de tu Comunidad Autónoma**																								
**Gobierno de tu Municipio**																								

(S9002) En una escala de 0 a 10, ¿qué nota pondrías a la gestión de la segunda oleada de coronavirus por parte de...?

Por favor, seleccione la respuesta apropiada para cada concepto:

	gestión sanitaria												gestión económica											
	No sabe / No contesta	0	1	2	3	4	5	6	7	8	9	10	No sabe / No contesta	0	1	2	3	4	5	6	7	8	9	10
**Gobierno de España**																								
**Gobierno de tu Comunidad Autónoma**																								
**Gobierno de tu Municipio**																								

(S9003) ¿Podrías indicarme a qué partido votaste en las últimas Elecciones Generales?

Por favor seleccione **sólo una** de las siguientes opciones:

- No tenía edad para votar
- No tenía derecho a voto
- Abstención
- Voté en blanco
- PSOE
- PP
- VOX
- UNIDAS PODEMOS
- Cs
- ERC-SOBIRANISTES
- EN COMÚ PODEM
- JxCAT
- EAJ-PNV
- MÁS PAÍS-EQUO
- EH Bildu
- CUP-PR
- MÉS COMPROMÍS
- COALICIÓN CANARIA-NUEVA CANARIAS
- BNG
- NAVARRA SUMA
- Otros

(S9004) Para terminar, si hoy se celebrasen elecciones al Congreso, ¿a qué partido votarías?

Por favor seleccione **sólo una** de las siguientes opciones:

- No votaría
- No tengo derecho a voto
- PSOE
- PP
- VOX
- UNIDAS PODEMOS
- Cs
- ERC-SOBIRANISTES
- EN COMÚ PODEM
- JxCAT
- EAJ-PNV
- MÁS PAÍS-EQUO
- EH Bildu
- CUP-PR
- MÉS COMPROMÍS
- COALICIÓN CANARIA-NUEVA CANARIAS
- BNG
- NAVARRA SUMA
- Otros

Agradecemos sinceramente tu interés y colaboración

Por favor, **INVITA A TUS CONTACTOS A PARTICIPAR EN LA ENCUESTA** reenviándoles el enlace o compartiéndolo en tus redes sociales.

Desde **GIPEyOP**, Universidad de Valencia, valoramos y agradecemos enormemente tus opiniones y nos gustaría seguir escuchándote en futuras investigaciones. Si deseas participar y colaborar con nosotros en futuras encuestas, que tu voz sea tenida en cuenta y recibir los resultados de esta investigación, puedes darte de alta rellenando cuatro datos en un formulario pulsando **aquí**.

Recibirás por correo electrónico un informe con los resultados provisionales del estudio si estás dado de alta. Un resumen de los resultados será enviado a los medios de comunicación.

Si Ud. se da de alta, de conformidad con lo establecido en la Ley Orgánica 3/2018, de Protección de Datos Personales y Garantía de los Derechos Digitales, sus datos se incluirán en un fichero titularidad de la Universidad de Valencia con las finalidades anteriormente indicadas. Siempre podrá ejercer sus derechos de acceso, rectificación, cancelación u oposición, solicitando la baja del fichero, visitando la web www.epo-uv.es o mediante escrito adjuntando documento identificativo a: Protección de Datos – Servei d'Informàtica – Universitat de València. C/ Amadeo de Saboya, 4. 460010, Valencia.
