# Supplementary material for: Dataset of the COVID-19 post-lockdown survey conducted by GIPEyOP in Spain
Source: Data Brief. 2021 Dec 24;40:107763. doi: 10.1016/j.dib.2021.107763 (PMC8704783; doi:10.1016/j.dib.2021.107763)
Supplement: Supplementary file 3 [file mmc3.pdf]

COVID-19 Lockdown Survey (autumn)

Participating in this survey is **voluntary** and **confidential** (LO 3/2018 on Protection of Personal Data and Guarantee of Digital Rights). All responses are anonymous, and the conclusions will be presented in aggregate form. The survey will remain open until **October 15**.

You can avoid answering any question you want or don't know the answer to. The estimated time to answer the survey is **10 minutes**.

To advance in the survey go to the bottom of the page and click Next.

Thanks to citizen collaboration, from GIPEyOP we are managing to improve the analysis methodologies and thereby be able to be useful, offering **quality**, **objective**, and **credible** results. We want you to help us continue moving in that direction. For more information, to know the conditions in which this survey is framed or about those responsible for it, click **here**.

We would appreciate INVITE YOUR CONTACTS TO PARTICIPATE IN THE SURVEY, by forwarding the link or sharing it on your social networks.

There are 40 questions in the survey

Section I (demographic variables)

(S1001) Please select the province in which you currently reside

Please select **only one** of the following options:

- A Coruña
- Albacete
- Alicante/Alacant
- Almería
- Araba/Álava
- Asturias
- Ávila
- Badajoz
- Barcelona
- Bizkaia
- Burgos
- Cáceres
- Cádiz
- Cantabria
- Castellón/Castelló
- Ceuta
- Ciudad Real
- Córdoba
- Cuenca
- Gipuzkoa
- Girona
- Granada
- Guadalajara
- Huelva
- Huesca
- Illes Balears
- Jaén
- La Rioja
- Las Palmas
- León
- Lleida
- Lugo
- Madrid
- Málaga
- Melilla
- Murcia
- Navarra
- Ourense
- Palencia
- Pontevedra
- Salamanca
- Santa Cruz de Tenerife
- Segovia
- Sevilla
- Soria
- Tarragona
- Teruel
- Toledo
- Valencia/València
- Valladolid
- Zamora
- Zaragoza

(S1002) Could you tell us the size of the municipality in which you reside?

Please select **only one** of the following options:

- Less than 2,000 inhabitants
- Between 2,001 and 10,000
- Between 10,001 and 50,000
- Between 50,001 and 100,000
- Between 100,001 and 400,000
- Between 400,001 and 1,000,000
- More than 1,000,000 inhabitants

(S1003) Gender

Please select **only one** of the following options:

- Male
- Female

(S1004) Could you indicate your year of birth?

(enter four digits)

Your answer must be between 1919 and 2003

Only an integer value can be entered in this field.

Please write your answer here:

- ________

(S1005) Could you tell us your studies?

Please select **only one** of the following options:

- Without studies
- Primary education
- Secondary education
- Job training
- Baccalaureate
- University studies

Section II (daily life)

(S2001) At the moment, is the place where you live the same residence in which you were confined during the state of alarm (March 15 to June 21)?

Please select **only one** of the following options:

- Yes, it is my usual home.
- Yes, although it is not my usual residence.
- Yes, I would like to change, but I have no choice.
- No, I have moved to another residence.
- No, I have returned to my usual residence.

(S2002) How many people do you live with?

Please select **only one** of the following options:

- None
- One
- Two
- Three
- Four
- Five or more

(S2002A) Could you please indicate the number of dependents you live with?

(sick people, young people, minors, dependents, …)

Only answer this question if the following conditions are true:

The answer was NOT 'None' in question (S2002)

Please select **only one** of the following options:

- None
- One
- Two
- Three
- Four
- Five or more

(S2002B) Could you please indicate the number of high-risk people you live with?

(old people, pregnant, sanitary, cops, cashiers, …)

Only answer this question if the following conditions are true:

The answer was NOT 'None' in question (S2002)

Please select **only one** of the following options:

- None
- One
- Two
- Three
- Four
- Five or more

Section III (employment situation)

(S3001) Could you mark how is your employment situation? Compared to…

Please select the appropriate answer for each item:

	the alarm state	before the alarm state
**Same**		
**Better**		
**Worse**		

(S3002) Could you mark which of the following activity profiles you are currently in?

Please select **only one** of the following options:

- I am salaried employee and telework
- I am salaried employee and I leave home to work
- I am salaried employee and I combine telework and work outside the home
- I am self-employed, and telework
- I am self-employed, and I leave home to work
- I am self-employed, and I combine telework and work outside the home
- I am self-employed without no possibility of practising my profession
- I am in an ERTE (Temporary Lay-off Plan)
- I was fired after the state of alarm period
- I was fired during the state of alarm period
- Sick leave/pregnancy
- I am unemployed or on leave of absence
- Retired
- Student
- Unpaid work at home
- I work without a contract outside the home
- Other

(S3003) Complete the sentence: I work in…

Only answer this question if the following conditions are true:

The answer was 'I am salaried employee and telework' or 'I am salaried employee and I leave home to work' or 'I am salaried employee and I combine telework and work out home' in question (S3002).

Please select **only one** of the following options:

- …the private sector
- …the public sector
- …both sectors

(S3003A1) Regarding the state of alarm, do you feel that your productivity at work has been affected after the end of the state of alarm?

Only answer this question if the following conditions are true:

The answer was 'I am salaried employee and telework' or 'I am salaried employee and I leave home to work' or 'I am salaried employee and I combine telework and work out home' or 'I am self-employed, and telework' or 'I am self-employed, and I leave home to work' or 'I am self-employed, and I combine telework and work out home' or 'I work without a contract outside my home' in question (S3002).

Please select **only one** of the following options:

- Yes, I have a higher performance.
- Yes, I have a lower performance.
- No.

(S3003A2) Do you think your work is threatened by this second wave of infections?

(you can indicate more than one option)

Only answer this question if the following conditions are true:

The answer was 'I am salaried employee and telework' or 'I am salaried employee and I leave home to work' or 'I am salaried employee and I combine telework and work out home' or 'I am self-employed, and telework' or 'I am self-employed, and I leave home to work' or 'I am self-employed, and I combine telework and work out home' or 'I work without a contract outside my home' in question (S3002).

Please check the options that apply:

- Yes, because of a lack of economic activity due to the crisis.
- Yes, due to staff cuts.
- Yes, due to salary cuts.
- Yes, because of having had to help in the family environment and having underperformed at work.
- No, everything will stay the same.

(S3002B) What is your experience of working at home after the end of the state of alarm?

(you can indicate more than one option)

Only answer this question if the following conditions are true:

The answer was 'I am salaried employee and telework' or 'I am salaried employee and I combine telework and work out home' or 'I am self-employed, and telework' or 'I am self-employed, and I combine telework and work out home' in question (S3002).

Please check the options that apply:

- I am making better use of my time than in my place of work.
- It is difficult to reconcile work and family life.
- I wouldn't mind continuing to telework.
- I prefer to commute to my place of work.
- I would like to alternate between the two options.
- I was already teleworking before the state of alarm.

(S3002C1) Are you concerned about how the 20-21 course will go?

Only answer this question if the following conditions are true:

The answer was 'Student' in question (S3002).

Please select **only one** of the following options:

- Yes, I am afraid of being in the classroom and catching the virus.
- Yes, I am not sure how the classes will be taught.
- Yes, I have not received enough information.
- Yes, I do not have the conditions to study from home if necessary.
- Yes, for other reasons.
- I have no concerns.

(S3002C2) How are you going to cover the costs of university studies?

(you can indicate more than one option)

Only answer this question if the following conditions are true:

The answer was 'Student' in question (S3002).

Please check the options that apply:

- My family.
- I have started to work after the end of state of alarm.
- I was already working before the state of alarm starts.
- I have savings.
- I have applied for a scholarship due to lack of resources.
- Others.

Section IV (household chores)

(S4001) For each activity, leave the box blank if not applicable or mark with 0, 1, 2, 3, 4, 5, 6 or 7 depending on the number of days per week you carried out / carry out the following activities

	before lockdown	during lockdown	after lockdown
Preparing midday meal	☐	☐	☐
Cleaning the bathroom	☐	☐	☐
Helping with children's homework	☐	☐	☐
Playing with minors	☐	☐	☐
Preparing the dinner	☐	☐	☐
Bathing dependent persons	☐	☐	☐
Leaving the house to look after other dependents	☐	☐	☐
Washing up after meals	☐	☐	☐
Dusting	☐	☐	☐
Cleaning the floor	☐	☐	☐
Going out for grocery shopping	☐	☐	☐
Doing the washing	☐	☐	☐
Ironing	☐	☐	☐
Throwing out the rubbish	☐	☐	☐

(S4002) How many days of the week did / do you have outside help to carry out household chores?

	Weekly frequency
Before lockdown	☐
After lockdown	☐

Section V (fears and cares)

(S5001) Let's talk about leaving the house after confinement

Please select **only one** of the following options:

- I have practically not gone out, but I am not afraid.
- I have practically not gone out because I am afraid.
- I go out alone to carry out basic tasks (walking the dog, shopping, work, care ...), although I am afraid.
- I go out alone to carry out basic tasks (walking the dog, shopping, work, care ...), and I am not afraid.
- I go out normally and I am not afraid.
- I go out normally, but I am a bit afraid.

(S5002) In general, with respect to before the beginning of this crisis that we are experiencing, how are you sleeping?

Please select **only one** of the following options:

- Same
- Better
- Worse

(S5003) In the current situation, which of the following measures are you following?

(you can indicate more than one option)

Please check the options that apply:

- I change the mask after its useful life.
- I wear a mask, but I reuse it more than I should.
- When I take off my mask I am careful where I keep it.
- I sanitize my hands whenever I touch something (public transport, coins…).
- I take care of my immune system (food, vitamin supplements, physical exercise…).
- I do not follow any special measures, I wear a mask out of obligation.

Section VI (holidays)

(S6001) What did you do last summer, 2019?

(you can indicate more than one option)

Please check the options that apply:

- Worked. I didn't have any holiday.
- Worked and enjoyed a few days of holiday.
- Do not travel due to lack of financial resources.
- Went to the countryside, to my second residence.
- Went to the beach, to my second residence.
- Went to the mountains, to my second residence.
- Went on a trip in Spain.
- Went on a trip outside of Spain.
- Others.

(S6002) How long have you had on vacation after the state of alarm?

Please select **only one** of the following options:

- I have had no holiday time.
- Less than a week.
- About fifteen days.
- Between fifteen days and a month.
- More than one month.

(S6002A) What have you done during the holidays that you have had after the state of alarm?

(you can indicate more than one option)

Only answer this question if the following conditions are true:

The answer was 'More than one month' o 'Between fifteen days and a month' o 'About fifteen days' o 'Less than a week' in question (S6002).

Please check the options that apply:

- Did not travel due to lack of financial resources.
- Did not travel due to economic uncertainly.
- Did not travel due to fear of contagion.
- Went to the countryside, to my second residence.
- Went to the beach, to my second residence.
- Went to the mountains, to my second residence.
- Went on a trip in Spain.
- Going on a trip outside of Spain.
- Others.

(S6002B) How have you managed the holidays?

Only answer this question if the following conditions are true:

The answer was 'Go to the country, to my second residence' or 'Go to the beach, to my second home' or 'Go to the mountains, to my second residence' or 'Go on a trip through Spain' or 'Going on a trip outside of Spain' in question (S6002A).

Please select **only one** of the following options:

- I have not needed to make any arrangements.
- By phone.
- By Internet.

(S6002C) Have you been afraid of COVID-19 on your vacation?

(you can indicate more than one option)

Only answer this question if the following conditions are true:

The answer was 'Go to the country, to my second residence' or 'Go to the beach, to my second home' or 'Go to the mountains, to my second residence' or 'Go on a trip through Spain' or 'Going on a trip outside of Spain' in question (S6002A).

Please check the options that apply:

- Yes, people did not wear masks
- Yes, people did not keep a safe distance
- Yes, there was an outbreak in a nearby location
- Yes, in my local environment there was a contagion
- Yes, around me were high risk people
- I was afraid out of respect for the disease
- I was not afraid

Section VII (social life)

(S7001) Regarding the number of people you interact with, have you changed your habits as a result of COVID?

(you can indicate more than one option)

Please check the options that apply:

- No, same as before
- Yes, I only socialise with the people I live with
- Yes, I only socialise with the people within my closest circle
- Yes, I have reduced the number of people I interact with

(S7002) Have you changed your habits when you leave your usual residence?

Please select **only one** of the following options:

- I only choose open spaces
- I might choose closed spaces as long as there is good ventilation
- I usually avoid closed spaces
- I do not restrict my choices

(S7003) How often did / do you use to go to a bar or restaurant?

Please select the appropriate answer for each item:

	Before the state of alarm	After the state of alarm
**Every day**		
**5 o 6 days a week**		
**3 o 4 days a week**		
**1 o 2 days a week**		
**Never**		

(S7004) How often did / do you play sports?

Please select the appropriate answer for each item:

	Before the state of alarm	After the state of alarm
**Every day**		
**5 o 6 days a week**		
**3 o 4 days a week**		
**1 o 2 days a week**		
**Never**		

Section VIII (digital divide)

(S8001) Compared to before the start of the pandemic, have you increased the use of internet and / or mobile?

(you can indicate more than one option)

	Due to work	Due to personal issues
**No, same as before**		
**Yes, more video conferencing**		
**Yes, more emails**		
**Yes, more use of Twitter**		
**Yes, more use of Facebook**		
**Yes, more use of WhatsApp**		
**Yes, more use of Telegram**		
**Yes, more use of Instagram**		

(S8002) From the list below, could you check the apps you use / used?

(you can indicate more than one option)

	Before the pandemic	Currently
**Facebook**		
**Instagram**		
**WhatsApp**		
**Telegram**		
**Twitter**		
**YouTube**		
**E-mail (to personal use)**		
**E-mail (to work)**		
**Skype**		
**Zoom**		
**Tams**		
**TikTok**		
**Tinder**		
**Others**		

(S8003) What device do you usually use to connect to the internet?

Please select **only one** of the following options:

- Smartphone
- Tablet
- Laptop
- PC
- Smartwatch
- Others

(S8004) In general, how do you prefer to communicate when you are not communicating in person?

Please select the appropriate answer for each item:

	to work	for personal motives
**Telephone call using a landline**		
**Telephone call using a mobile phone**		
**Message written by WhatsApp or Telegram**		
**Written email**		
**Audio message by WhatsApp or Telegram**		
**Videoconference (WhatsApp, Skype, Zoom ...)**		

(S8005) Do you usually have problems with your internet connection?

Please select **only one** of the following options:

- No
- Yes, the network goes down sometimes
- Yeah the network crashes
- Yes, the network is slow

Section IX (political management)

(S9001) On a scale of 0 to 10, what grade would you give the management of this crisis in the…?

Please select the appropriate answer for each item:

	Health management												Economic management											
	Don't know	0	1	2	3	4	5	6	7	8	9	10	Don't know	0	1	2	3	4	5	6	7	8	9	10
**Government of Spain**																								
**Government of your Autonomous Community**																								
**Government of your Municipality**																								

(S9002) On a scale of 0 to 10, what grade would you give the management of the second wave of coronavirus by…?

Please select the appropriate answer for each item:

	Health management												Economic management											
	Don't know	0	1	2	3	4	5	6	7	8	9	10	Don't know	0	1	2	3	4	5	6	7	8	9	10
**Government of Spain**																								
**Government of your Autonomous Community**																								
**Government of your Municipality**																								

(S9003) Could you tell me which party you voted for in the last General Election?

Please select **only one** of the following options:

- I was not old enough to vote
- I did not have the right to vote
- Abstention
- I voted blank
- PSOE
- PP
- VOX
- UNIDAS PODEMOS
- Cs
- ERC-SOBIRANISTES
- EN COMÚ PODEM
- JxCAT
- EAJ-PNV
- MÁS PAÍS-EQUO
- EH Bildu
- CUP-PR
- MÉS COMPROMÍS
- COALICIÓN CANARIA-NUEVA CANARIAS
- BNG
- NAVARRA SUMA
- Others

(S9004) Finally, if Congress elections were held today, which party would you vote for?

Please select **only one** of the following options:

- I would not vote
- I don’t have the right to vote
- PSOE
- PP
- VOX
- UNIDAS PODEMOS
- Cs
- ERC-SOBIRANISTES
- EN COMÚ PODEM
- JxCAT
- EAJ-PNV
- MÁS PAÍS-EQUO
- EH Bildu
- CUP-PR
- MÉS COMPROMÍS
- COALICIÓN CANARIA-NUEVA CANARIAS
- BNG
- NAVARRA SUMA
- Others

We sincerely appreciate your interest and collaboration

Please **INVITE YOUR CONTACTS TO PARTICIPATE IN THE SURVEY** by forwarding the link or sharing it on your social networks.

From **GIPEyOP**, University of Valencia, we value and greatly appreciate your opinions and we would like to continue listening to you in future research. If you want to participate and collaborate with us in future surveys, that your voice is considered and receive the results of this research, you can sign up by filling in four pieces of information in a form by clicking **here**.

You will receive by email a report with the provisional results of the study if you are discharged. A summary of the results will be sent to the media.

If you register, in accordance with the provisions of Organic Law 3/2018, on the Protection of Personal Data and Guarantee of Digital Rights, your data will be included in a file owned by the University of Valencia for the purposes above indicated. You can always exercise your rights of access, rectification, cancellation or opposition, requesting the cancellation of the file, visiting the website www.epo-uv.es or by writing attaching an identification document to: Data Protection - Servei d'Informàtica - Universitat de València . C / Amadeo de Saboya, 4. 460010, Valencia.
